# β-catenin ameliorates myocardial infarction by preventing YAP-associated apoptosis

**DOI:** 10.1016/j.clinsp.2023.100189

**Published:** 2023-04-02

**Authors:** Haofei Kang, Weiwei Jiang

**Affiliations:** a Department of Cardiovascular Medicine, Yantaishan Hospital, Yantai, China; b Department of Cardiovascular Medicine, The 970th Hospital of the Joint Logistic Support Force of the People's Liberation Army, Yantai, China

**Keywords:** β-catenin, Cardiomyocytes, Heart failure, Myocardial Infarction, Yes-associated protein (YAP)

## Abstract

**Objective:**

To explore whether the effect of β-catenin on MI and MI-induced cardiomyocyte apoptosis is YAP-dependent.

**Methods:**

The authors established an MI rat model by ligating the anterior descending branch of the left coronary artery, and an MI cell model by treating cardiomyocytes with H_2_O_2_.

**Results:**

β-catenin downregulation was observed in MI cardiac tissues and in H_2_O_2_-treated cardiomyocytes. Lentiviral-CTNNB1 was administered to MI rats to upregulate β-catenin expression in MI cardiac tissue. β-catenin recovery reduced the myocardial infarct area, fibrosis, and apoptotic cell death in MI rats. H_2_O_2_ treatment attenuated cell viability and induced cell death in cardiomyocytes, whereas β-catenin overexpression partially reversed these changes. Moreover, H_2_O_2_ treatment caused the deactivation of Yes-Associated Protein (YAP), as detected by increased YAP phosphorylation and reduced the nuclear localization of YAP. Upregulation of β-catenin expression reactivated YAP in H_2_O_2_-treated cardiomyocytes. Reactivation of YAP was achieved by administration of Mitochonic Acid-5 (MA-5) to H_2_O_2_-treated cardiomyocytes, and deactivation of YAP by CIL56 treatment in β-catenin-overexpressing H_2_O_2_-treated cardiomyocytes. MA-5 administration increased cell viability and repressed apoptosis in H_2_O_2_-treated cardiomyocytes, whereas CIL56 treatment counteracted the effects of β-catenin overexpression on cell survival and apoptosis.

**Conclusions:**

The present data indicate that β-catenin and YAP are effective treatment targets for MI, blocking the apoptotic death of cardiomyocytes.

## Highlights

• β-catenin protects cardiomyocytes against H_2_O_2_-induced apoptosis.
• CIL56 restored apoptotic cell death in H_2_O_2_-treated cardiomyocytes, reversing the effects of β-catenin.
• The β-catenin/YAP axis may be a target for the treatment of acute MI.

## Introduction

Acute Myocardial Infarction (MI), driven by coronary artery occlusion, is one of the major triggers of cardiovascular incidence and mortality worldwide. Acute MI can result in adverse cardiovascular outcomes following myocardial ischemia generation, circulatory arrest in coronary heart disease, and cardiac surgery.^1^ Despite this, the relevant mechanisms underlying MI injury are intricate and poorly explored.

Membrane-bound catenin can maintain cell polarity and tissue structure at adhesive junctions by connecting the actin cytoskeleton with the cadherin cytoplasmic tail.^2^ Cytoplasmic catenin translocates to the nucleus, where it forms part of a complex containing Tcf/Lef transcription factors and initiates the expression of specific genes relevant to cell survival and proliferation.^3^ The essential roles of β-catenin in neoplastic diseases have already been well described;^4^, ^5^, ^6^ comparably less is known about the impact of β-catenin on cardiomyocytes during MI development. Cytoplasmic β-catenin has been detected in the endothelial cells of pre-existing and freshly generated blood vessels in the infarction area in the first week following MI.^3^ Hahn et al. demonstrated that in a rat MI model, injecting the infarct border region with adenovirus-β-catenin could lead to a dramatically reduced MI size, with both anti-apoptotic effects and cell cycle initiation in myofibroblasts and cardiomyocytes.^7^ Therefore, β-catenin may be an attractive strategy for slowing or reducing the pathogenic changes associated with various forms of heart failure.

The Hippo pathway is a highly conserved tumor inhibitor pathway that is primarily composed of mammalian MST1/2 and Large Tumor Suppressor Kinase (LATS) 1/2, Yes-Associated Protein (YAP), and its paralog transcriptional co-activator with PDZ-binding motif (TAZ). MST1/2 and LATS1/2 are oncosuppressive kinases. Once pathway activation occurs, LATS1/2 is phosphorylated and activated by MST1/2, in turn, LATS1/2 phosphorylates YAP and suppresses its activity.^8^ Hippo pathway inactivation is closely linked to the occurrence and progression of various tumors.^9^ YAP and TAZ are essential for cardiac regeneration and repair.^10^ Mice lacking epicardial YAP and TAZ display severe myocardial fibrosis and pericardial inflammation after MI, leading to cardiomyopathy and death.^11^ YAP overexpression in embryonic rat hearts can enlarge heart size and facilitate cardiac regeneration and contraction following MI by eliciting cardiomyocyte proliferation. YAP deletion hinders cardiomyocyte proliferation, resulting in myocardial hypoplasia and premature or embryonic lethality.^12^

YAP has been confirmed to have both positive and negative functions in Wnt/β-catenin signaling.^13^ Some studies have shown that Wnt/β-catenin signaling can activate YAP, which cooperates with MYC during liver transformation and mitogenic activation. In line with these modulatory functions, YAP accumulated through the activation of Myc/β-catenin and was required for the subsequent cell proliferation, as well as tumor cell growth and survival,^14^ suggesting that β-catenin acts as an upstream modulator of YAP during tumorigenesis. However, the effects and mechanisms of their crosstalk in MI are poorly understood. In this work, an H_2_O_2_-elicited cardiomyocyte injury cell model and an MI rat model were constructed to explore whether the effect of β-catenin on MI and MI-induced cardiomyocyte apoptosis is YAP-dependent. The present results indicate that both β-catenin and YAP may be promising targets for MI treatment.

## Materials and methods

### Animal protocols

Adult male Wistar rats (220±20g) were provided by the Viral River Animal Experimental Center. The study was approved by the ethics committee of the 970^th^ Hospital of the Joint Logistic Support Force of the People's Liberation Army and all animal experiments followed the ARRIVE guidelines, as per the Chinese Guidance of Humane Laboratory Animal Use guidelines. MI injuries were generated through the ligation of the Left Anterior Descending (LAD) coronary artery, as described by Yang et al.^15^ Briefly, rats were intraperitoneally injected with 100 mg/kg pentobarbital sodium for anesthesia, and their temperature was maintained at 37°C using a heating pad. After locating and exposing the left coronary artery, the artery was ligated for 45 min. Reperfusion was then initiated by the removal of the ligature. Sham-operated control rats underwent a similar operation, excluding the ligation of the left coronary artery. The muscle layer and skin were then closed, and rats were recuperated for three weeks after reperfusion prior to hemodynamic measurements. Postoperative pain was eased through an intramuscular injection of 0.65 mg/kg buprenorphine hydrochloride.

### Lentivirus construction

Pljm1-EGFP plasmid driven by CMV promoter was connected with CTNNB1 sequence. This vector also encodes Green Fluorescence Protein (GFP) for the selection of stably infected clones. The authors also accessed the successful overexpression of CTNNB1 in HEK293 cells (Invitrogen) by Taqman qPCR. Thereafter, CTNNB1 sequence was inserted into Pglv3/h1/gfp+puro plasmid vector (Genepharma, Shanghai) and driven by H1 promoter. The pseudoviral particles were produced using a lentivector packaging system (Genepharma, Shanghai) according to the manufacturer's instructions.

### Experimental groups

To evaluate the effect of CTNNB1 treatment on rats with MI, animals were intramyocardially injected with lentivirus containing CTNNB1 sequence in the border zone immediately after LAD ligation.^16^ A total of 20 µL virus (2.0×10^7^ viral particles) was injected into 3 regions of the border zone using a 29-gauge Hamilton syringe.

Twenty-four healthy wild-type rats were randomly divided into three groups with eight rats per group: (1) Sham operation control rats (control group); (2) LAD ligation rats (MI group); (3) Lentiviral (LV)-CTNNB1-transduced and LAD ligation rats (MI+LV-CTNNB1 group). In the last group, rats were treated with LV-CTNNB1 after undergoing the MI model protocol; otherwise, they underwent the same protocols as the MI group.

### Infarct size measurement

After 2h reperfusion, Sirius Red staining was used to measure infarct sizes. Briefly, rats were sacrificed, and their hearts were fixed with paraformaldehyde overnight. Hearts were then cut into 5 sections (1-mm thick), which were flat embedded with paraffin, and then further cut to sections of 4-μm thickness. These sections were then subjected to Triphenyl Tetrazolium Chloride (TTC) staining to measure the infarct volume, then placed on a light table and photographed on both sides. Different regions were subsequently delineated. Infarct sizes were calculated as the ratio of the infarct volume to the left ventricle wall volume.

### Hematoxylin and eosin (H&E) staining

Hearts were embedded in paraffin, cut into 4-μm sections, and subjected to H&E staining. Briefly, after xylene deparaffinization, alcohol rehydration, and hematoxylin staining, sections were differentiated with 1% acid alcohol and counterstained with eosin-phloxine. They were then mounted using a xylene-based mounting medium, and subsequently examined under a microscope for histological alterations, e.g., hydropic cardiomyocytes, hemorrhage, neutrophilic and lymphohistiocytic infiltrate, and acute myocardial necrosis.

### TUNEL staining

Apoptotic cells were labeled using the terminal deoxynucleotidyl transferase-mediated dUTP Nick-End lLabeling (TUNEL) fluorescent Fluorescein Isothiocyanate (FITC) kit (Roche, Germany), following the manufacturer's instructions. Subsequently, heart sections (7d post-MI) were immersed in Hoechst 33342 solution for cell nuclei staining. Fluorescent staining was observed using a FluoView FV1000 microscope (Olympus). The numbers of TUNEL-positive and total cells were analyzed using Image-Pro Plus.

### Quantitative PCR (qPCR)

TRIzol was used to isolate total RNA from cardiomyocytes and cardiac tissues. cDNA was obtained by reverse transcription of RNA using a cDNA Reverse Transcription kit (Vazyme Biotech Co., Ltd.), incubating for 1h at 42°C, then 5-min at 75°C. qPCR was conducted using the SYBR Green PCR Master kit (Vazyme Biotech Co., Ltd.). The thermocycling conditions were as follows: initial denaturation at 95°C for 3-min, and 40 cycles of 95°C for the 30s, 56°C for 30s, and 72°C for 30s. Fold changes in gene expression were calculated using the 2^−ΔΔCq^ method.^17^ mRNA expression levels were normalized to those of glyceraldehyde 3-phosphate dehydrogenase, the internal control. All experiments were performed in triplicate.

### Western blot (WB)

Protein was isolated from heart tissues using a radioimmunoprecipitation assay lysis buffer, according to the relevant protocol. Total protein concentrations were determined using a bicinchoninic acid protein assay kit. Protein specimens were treated with sodium dodecyl sulfate-polyacrylamide gel electrophoresis and then transferred to nitrocellulose membranes, which were then incubated overnight with the appropriate primary antibodies. Bound antibodies were visualized using horseradish peroxidase-conjugated secondary antibodies. Band intensities were quantified using BandScan 5.0 software.

### Cardiomyocyte culture

Cardiomyocytes were isolated from 6–8 week-old mice and cultured.^18^^,^^19^ Briefly, hearts were resected under sterile conditions, and ventricle specimens were cut into pieces, before digestion with 0.25% trypsin. Isolated cells were suspended in Dulbecco's Modified Eagle Medium (DMEM) with 10% Fetal Bovine Serum (FBS), centrifuged at 1000 rpm for 5 min, then resuspended in DMEM with 10% FBS and incubated for 120 min. Cells were then plated into non-coated culture flasks. Bromodeoxyuridine (0.1 mM) was added to the medium to clear non-myocytes. Cardiomyocytes were cultured at 37°C and 5% CO_2_.

### Cell transfection

The β-catenin overexpression vector pcDNA3.1-β-catenin (Genscript Co., Ltd.; Beijing, China) was used to transfect cardiomyocytes using Lipofectamine 3000, following the manufacturer's instructions. After 40h of transfection, cells were collected and used for subsequent experiments.

### Cell counting Kit-8 (CCK-8) assay

Cell survival was examined using a CCK-8 assay. Briefly, cells were incubated with a 10 μL CCK-8 solution for 120 min. Absorbance at 450 nm was measured using an Infinite M200 microplate reader.

### Colony formation assay (CFA)

Cardiomyocytes were inoculated into 6-well plates and cultured for 10d. Colonies were then fixed with 10% formaldehyde for 5 min and stained with 1% crystal violet for 30 sec.

### Flow cytometry (FCM)

Cardiomyocyte apoptosis was assessed using an Annexin V-FITC/PI apoptosis kit. Briefly, transfected cardiomyocytes were resuspended in 20 µL binding buffer, and incubated with 10 µL Annexin V-FITC and 5 µL Propidium Iodide (PI) for 20 min in the dark. Cell apoptosis was then analyzed by FCM.

### Statistical analysis

Results are displayed as the mean ± standard deviation. One-way analysis of variance followed by Tukey's post-hoc test, or Student's *t*-test, was used to identify significant differences between multiple groups and two groups, respectively. The threshold for significance was set as *p* < 0.05.

## Results

### β-catenin expression was reduced in the cardiac tissue of MI rats

**Fig. 1:**
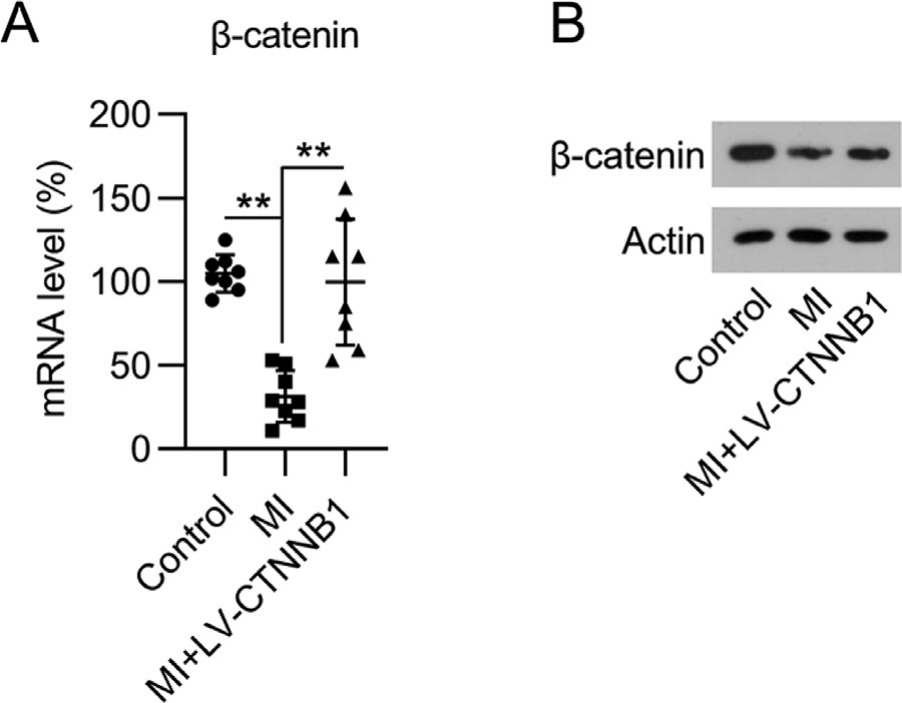
Expression of β-catenin in an MI rat model. A rat model of MI was established via the ligation of the LAD coronary artery, with or without LV-CTNNB1 transduction. (A) qPCR and (B) WB analysis of β-catenin mRNA and protein expression levels in the cardiac tissue from each group. LAD, Left Anterior Descending; LV, Lentiviral; MI, Myocardial Infarction; qPCR, quantitative PCR; WB, Western Blot.

To determine the impact of β-catenin during the development of MI, the authors first investigated its expression during MI development in rats. The authors established a LAD coronary artery ligation-induced MI rat model, and also transduced these animals with LV-CTNNB1. qPCR and WB analysis identified that β-catenin mRNA and protein expression levels were decreased in the cardiac tissues of the MI group compared to the control group, whereas LV-CTNNB1 transduction caused an elevation of β-catenin levels (Fig. 1A, B). β-catenin has been suggested to be negatively associated with the progression of MI.

### Exogenous expression of β-catenin ameliorated cardiac infarction in MI rats

**Fig. 2:**
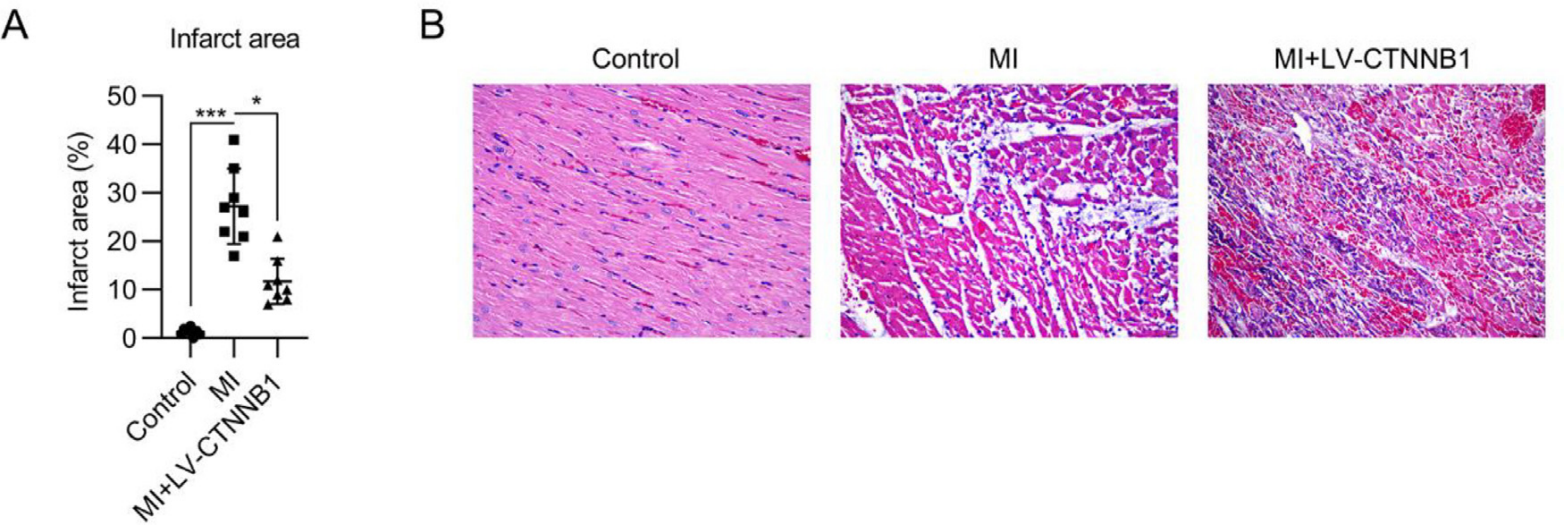
Effect of β-catenin upregulation on infarct size and pathological changes in the cardiac tissue of MI rats. A rat model of MI was established via the ligation of the LAD coronary artery, with or without LV-CTNNB1 transduction. (A) TTC staining of cardiac tissues from each group. The right panel shows the quantification of the infarct area. (B) Pathological changes in cardiac tissues were examined using H&E staining. H&E, Hematoxylin and Eosin; LAD, Left Anterior Descending; LV, Lentiviral; MI, Myocardial Infarction; TTC, Triphenyl Tetrazolium Chloride.

To evaluate the function of β-catenin in MI development, infarct size, and pathogenic changes were examined by TTC and H&E staining, respectively. TTC staining showed that the infarct area proportion in the MI group reached 28% following LAD coronary artery ligation, whereas upregulation of β-catenin decreased the infarct area to 12% (Fig. 2A).

H&E staining was used to identify pathogenic changes in the MI model. The cardiomyocytes in the anterior wall region of the left ventricle were disordered and decreased in number in the MI group. Pathogenic changes, including hypertrophy and fibrosis, were observed. These MI-induced changes were alleviated by the exogenous expression of β-catenin (Fig. 2B).

### LV-CTNNB1 transduction reduced myocardial cell apoptosis in MI rats

**Fig. 3:**
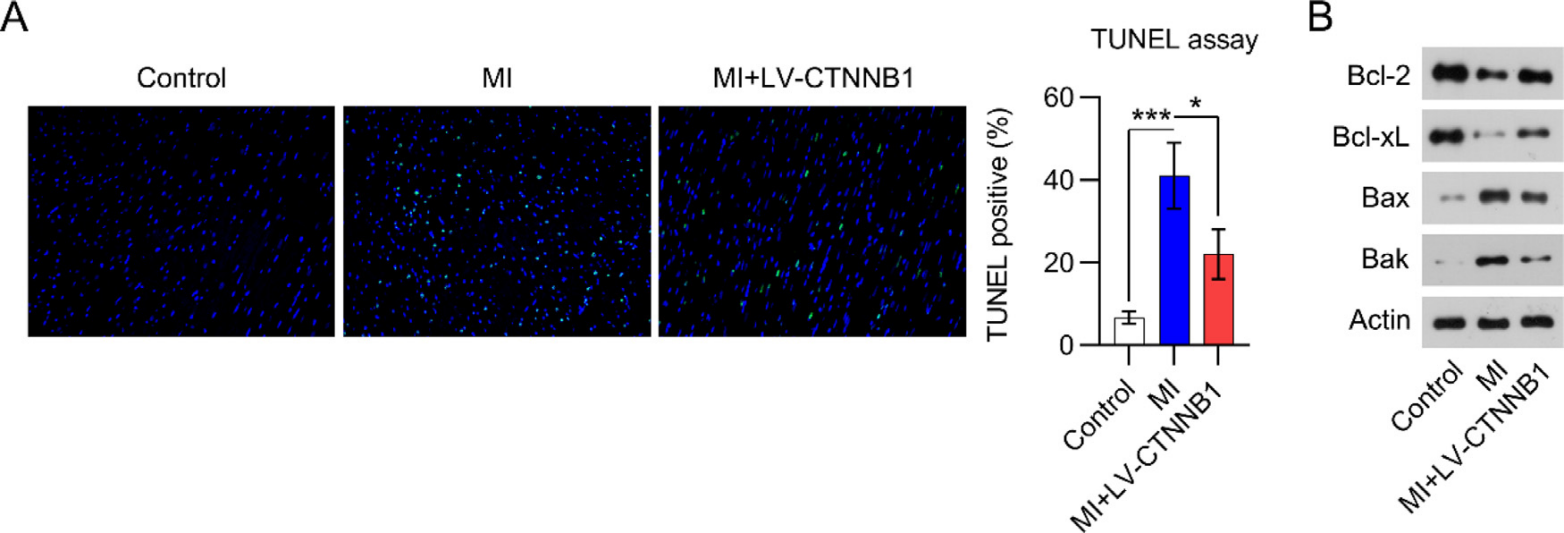
β-catenin upregulation reduced apoptosis in the cardiac tissue of MI rats. A rat model of MI was established via the ligation of the LAD coronary artery, with or without LV-CTNNB1 transduction. TUNEL staining was used to analyze apoptotic cells in the cardiac tissue of MI rats. LAD, Left Anterior Descending; LV, Lentiviral; MI, Myocardial Infarction; TUNEL, Terminal Deoxynucleotidyl Transferase-Mediated dUTP Nick-End Labeling; WB, Western Blot.

Apoptotic cell death in cardiac tissue is a major manifestation of acute and chronic MI. TUNEL staining was performed to detect apoptotic cardiomyocytes in the cardiac tissue of MI rats. Images show that the number of apoptotic cells was remarkably elevated in the cardiac tissues of the MI group relative to the control group. Even so, less apoptotic cells were observed in the MI+LV-CTNNB1 group than in the MI group (Fig. 3).

### β-catenin overexpression increased cell viability and inhibited apoptotic death in H_2_O_2_-treated cardiomyocytes

**Fig. 4:**
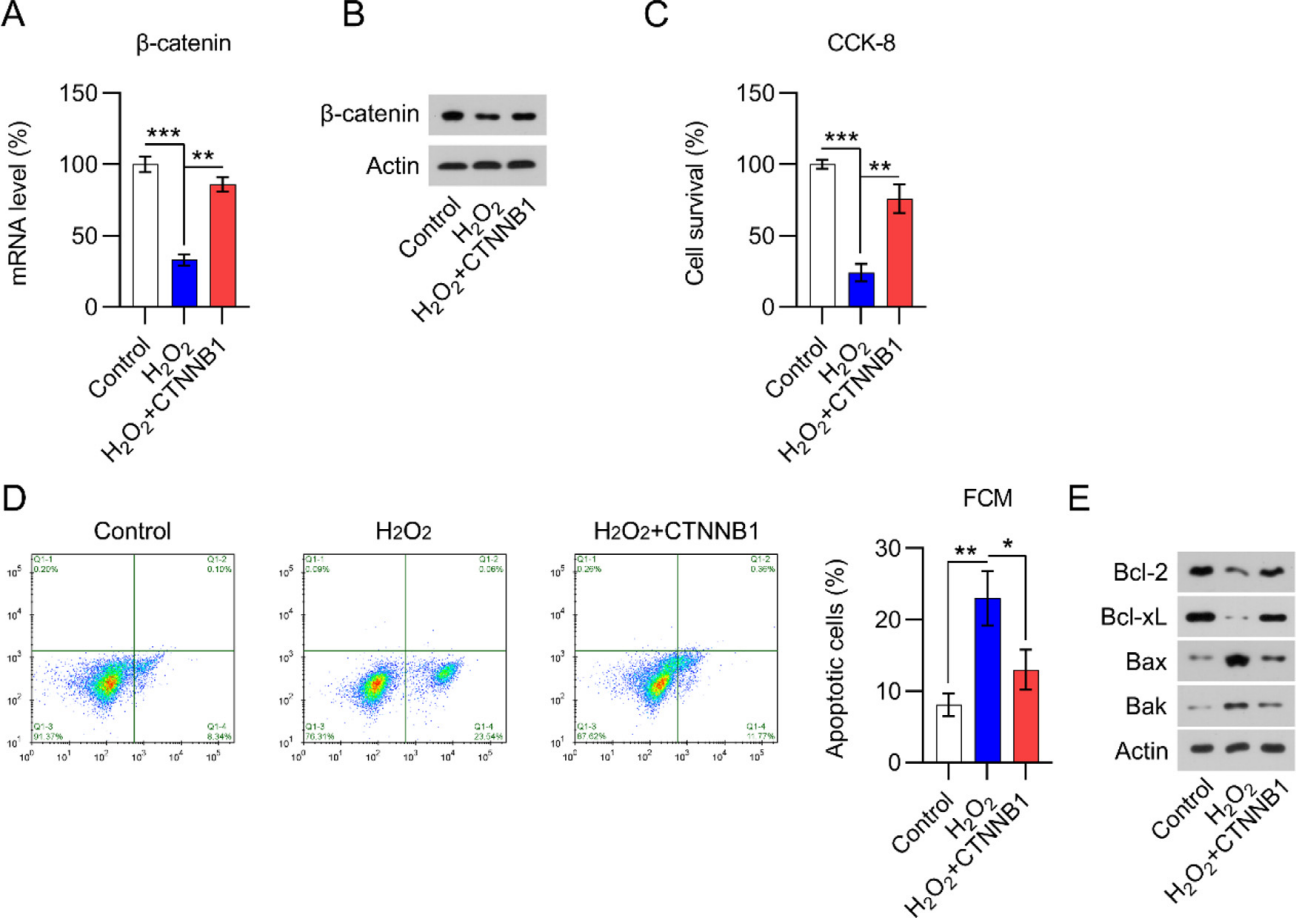
β-catenin was downregulated in H_2_O_2_-treated cardiomyocytes. Cardiomyocytes were first transfected with pcDNA3.1-CTNNB1 for 24h and then treated with 100 μM H_2_O_2_ for over 12h. (A) qPCR and (B) WB analysis of β-catenin mRNA and protein levels in cardiomyocytes with the indicated treatment. (C) CCK-8 assays showing cell growth rates and survival, respectively. (D) Annexin V-FITC and PI FCM analysis of the percentage of apoptotic cells. (E) WB analysis of Bax, Bak, Bcl-2, and Bcl-xL protein expression in cells. CCK-8, Cell Counting Kit-8; CFA, Colony Formation Assay; FCM, Flow Cytometry; FITC, Fluorescein Isothiocyanate; PI, Propidium Iodide; Qpcr, quantitative PCR; WB, Western Blot.

H_2_O_2_ is widely believed to be capable of inducing cardiomyocyte damage and death.^20^ Several studies have shown that H_2_O_2_ can result in cardiomyocyte damage in a concentration- and time-dependent manner.^18^ Thus, qPCR and WB were used to determine β-catenin levels in H_2_O_2_-treated and non-treated cardiomyocytes. A significant decrease in β-catenin expression was detected in H_2_O_2_-treated cells compared with expression in non-treated cells (Fig. 4A, B).

As H_2_O_2_ could suppress cardiomyocyte viability, the authors assessed whether the overexpression of β-catenin would counteract H_2_O_2_-induced cell damage. The efficacy of β-catenin overexpression in H_2_O_2_-induced cardiomyocytes was determined by qPCR and WB (Fig. 4A, B). CCK-8 (Fig. 4C) assays showed that H_2_O_2_-exposed cardiomyocyte viability and growth were dramatically rescued by β-catenin overexpression. The authors also hypothesized that β-catenin could protect cardiomyocytes from H_2_O_2_-mediated apoptotic death. In line with this hypothesis, Annexin V-FITC and PI staining FCM data demonstrated that β-catenin overexpression distinctly reduced H_2_O_2_-induced apoptosis (Fig. 4D). In addition, WB analysis of Bax, Bak, Bcl-2, and Bcl-xL protein expression in cardiomyocytes clearly showed that H_2_O_2_ induced Bax and Bak expression while reducing Bcl-2 and Bcl-xL expression (Fig. 4E). β-catenin upregulation counteracted the effect of H_2_O_2_ on Bax, Bak, Bcl-2, and Bcl-xL expression, suggesting that β-catenin protects cardiomyocytes against H_2_O_2_-induced apoptosis.

### β-catenin activated YAP in H_2_O_2_-treated cardiomyocytes

β-catenin acts as an upstream modulator of YAP in tumor cells.^8^ Therefore, the authors next investigated the role of β-catenin in the regulation of YAP activation in H_2_O_2_-induced cardiomyocytes.

**Fig. 5:**
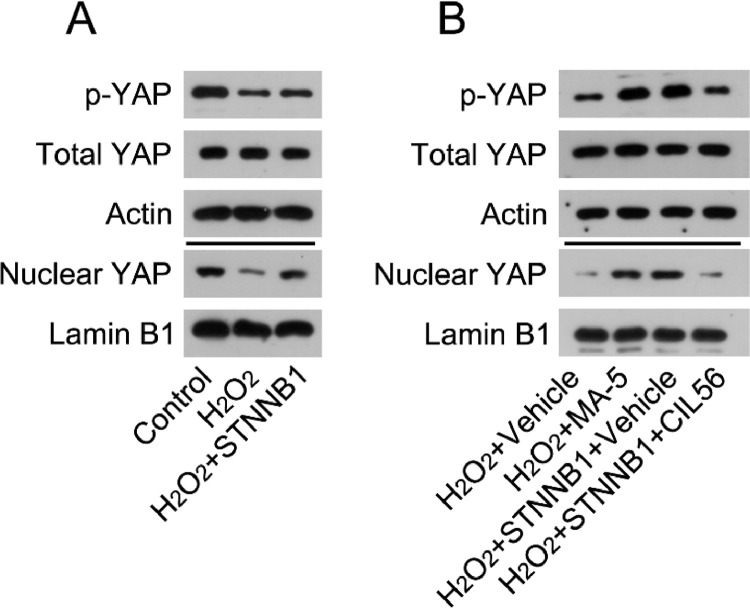
β-catenin reactivated YAP in the cardiac tissues of MI rats and H_2_O_2_-treated cardiomyocytes. (A) Cardiomyocytes were first transfected with pcDNA3.1-CTNNB1 for 24h, and then treated with 100 μM H_2_O_2_ for over 12h. Levels of total YAP, phosphorylated YAP, and nuclear-localized YAP in cardiomyocytes was analyzed by WB. (B) Cardiomyocytes were treated with 5 μM MA-5 for 12h and then treated with 100 μM H_2_O_2_ for over 12h. Cardiomyocytes transfected with pcDNA3.1-CTNNB1 were treated with 3.3 μM CIL56 for 12h and then treated with 100 μM H_2_O_2_ for over 12h. Levels of total YAP, phosphorylated YAP, and nuclear-localized YAP were analyzed by western blot. LAD, Left Anterior Descending; LV, Lentiviral; MA-5, Mitochonic Acid-5; MI, Myocardial Infarction; WB, Western Blot; YAP, Yes-Associated Protein.

The authors first determined the activation state of YAP in H_2_O_2_-treated cardiomyocytes. WB analysis indicated that H_2_O_2_ treatment caused YAP phosphorylation and reduced the nuclear levels of YAP. Furthermore, YAP was reactivated in H_2_O_2_-treated cardiomyocytes overexpressing β-catenin (Fig. 5A).

Next, the authors determined the involvement of YAP in β-catenin-regulated cell survival and death in H_2_O_2_-treated cardiomyocytes. H_2_O_2_-treated cardiomyocytes were treated with YAP activator Mitochonic Acid-5 (MA-5) for over 12h; H_2_O_2_-treated cardiomyocytes with β-catenin overexpression were treated with CIL56 for over 12h. WB analysis determined that MA-5 administration induced YAP activation in H_2_O_2_-treated cardiomyocytes, whereas CIL56 treatment caused YAP deactivation in β-catenin-overexpressing H_2_O_2_-treated cardiomyocytes (Fig. 5B).

### YAP reactivation counteracted the effects of β-catenin on the viability of cardiomyocytes

**Fig. 6:**
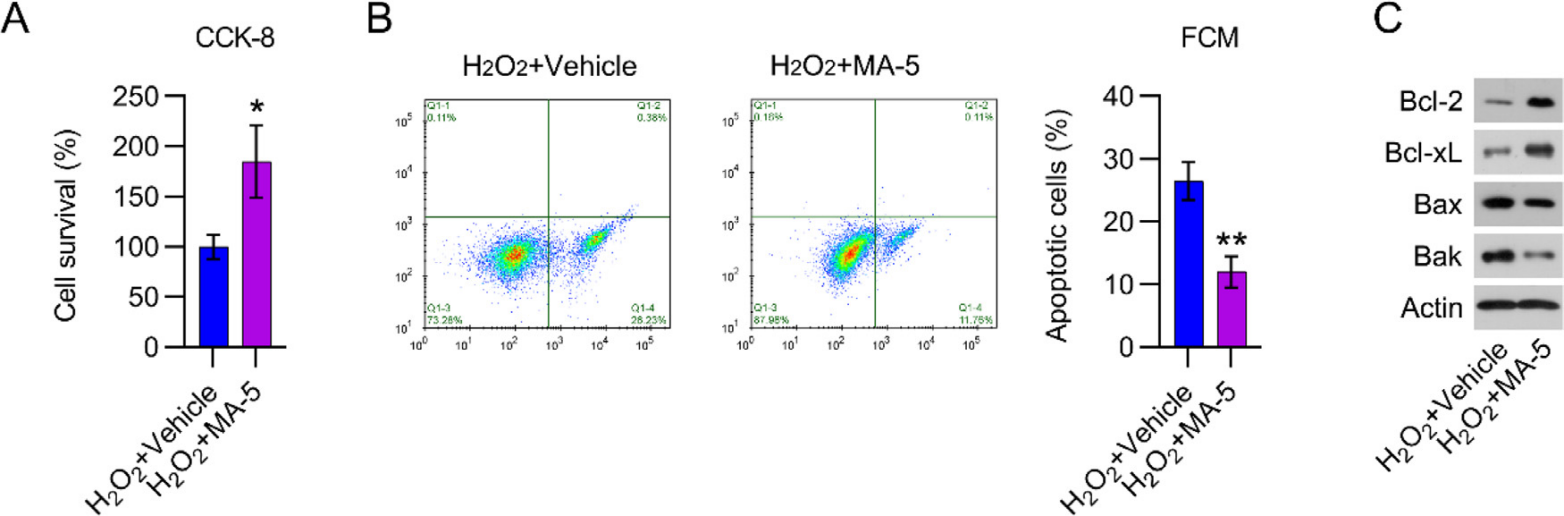
MA-5 administration reduced cell apoptosis in H_2_O_2_-treated cardiomyocytes. Cardiomyocytes were treated with 5 μM MA-5 for 12h and then treated with 100 μM H_2_O_2_ for over 12h. (A) CCK-8 assays were used to determine cell growth rates and survival, respectively. (B) Annexin V-FITC and PI FCM were performed to assess the percentage of apoptotic cells. (C) WB analysis of Bax, Bak, Bcl-2, and Bcl-xL protein expression in cells. CCK-8, Cell Counting Kit-8; CFA, Colony Formation Assay; FCM, Flow Cytometry; FITC, Fluorescein Isothiocyanate; MA-5, Mitochonic Acid-5; PI, Propidium Iodide; WB, Western Blot.

To examine the effects of YAP reactivation in H_2_O_2_-treated cardiomyocytes, cell viability, and death were analyzed. MA-5 administration resulted in greater cell viability than in cells treated with H_2_O_2_ only (Fig. 6A). H_2_O_2_-induced apoptosis in cardiomyocytes was also significantly reduced by MA-5 treatment, as assessed by FCM (Fig. 6B). In addition, Bax and Bak expression in H_2_O_2_-treated cells was decreased, whereas Bcl-2 and Bcl-xL levels were increased, in response to MA-5 treatment (Fig. 6C).

**Fig. 7:**
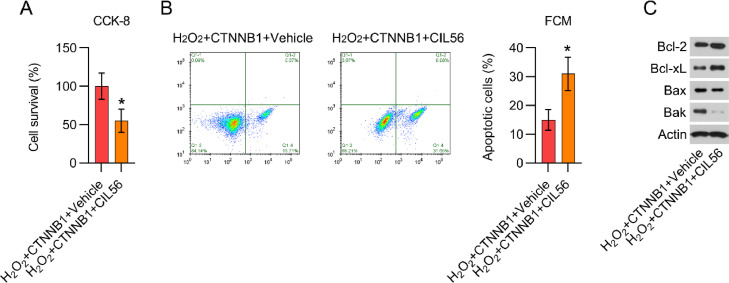
CIL56 increased cell apoptosis in β-catenin-overexpressing and H_2_O_2_-treated cardiomyocytes. Cardiomyocytes transfected with pcDNA3.1-CTNNB1 were treated with 3.3 μM CIL56 for 12h and then treated with 100 μM H_2_O_2_ for over 12h. (A) CCK-8 assays were used to determine cell growth rates and survival, respectively. (B) Annexin V-FITC and PI FCM were performed to assess the percentage of apoptotic cells. (C) WB analysis of Bax, Bak, Bcl-2, and Bcl-xL protein expression in cells. CCK-8, Cell Counting Kit-8; CFA, Colony Formation Assay; FCM, Flow Cytometry; FITC, Fluorescein Isothiocyanate; PI, Propidium Iodide; WB, Western Blot.

The authors further used CIL56 to deactivate YAP in β-catenin-overexpressing and H_2_O_2_-treated cardiomyocytes. CFA and CCK-8 assays showed that cell growth of β-catenin-overexpressing and H_2_O_2_-treated cardiomyocytes were attenuated by CIL56 exposure (Fig. 7A). FCM data demonstrated that the proportion of apoptotic cells was elevated by CIL56 (Fig. 7B). Additionally, the authors observed that CIL56 treatment increased Bax and Bak protein expression, but reduced Bcl-2 and Bcl-xL expression, in β-catenin-overexpressing and H_2_O_2_-treated cardiomyocytes (Fig. 7C), indicating that CIL56 restored apoptotic cell death in H_2_O_2_-treated cardiomyocytes, reversing the effects of β-catenin.

## Discussion

MI is a major cause of death worldwide. The current standardized therapy is reperfusion, which can paradoxically lead to severe injuries to the myocardium, such as cytokine generation and the production of reactive oxygen species. Accumulating studies and clinical trials have suggested that there are no efficient strategies to shield the myocardium from injuries caused by reperfusion. Apoptosis, a major factor contributing to cardiomyocyte dysfunction,^21^, ^22^, ^23^ plays a critical role in MI injury. To explore the relevant molecular mechanisms linking heart disease and cardiomyocyte apoptosis, it is necessary to elucidate which molecules serve as triggers for apoptosis; the determination of these molecules could also help identify potential effective therapeutic targets for MI. This study demonstrated that the induction of MI in a rat model activated the myocardial β-catenin signal transduction pathway and induced myocardial apoptosis, whereas exogenous expression of β-catenin attenuated apoptotic cell death in both animal and cell models of MI. β-catenin also impacted YAP activation. The cardioprotective role of β-catenin in MI may be due to the suppression of apoptosis in a YAP-dependent manner.

Matthijs-Blankesteijn et al. detected β-catenin in the endothelial cell cytoplasm of freshly generated vessels around the infarction area 4 and 7 days following MI. In contrast, no detectable levels of β-catenin were observed in the cytoplasm of vascular endothelial cells in non-infarcted areas.^3^ Hahn et al. explored the role of β-catenin in cardiac fibroblasts and cardiomyocytes, and whether β-catenin overexpression was capable of decreasing MI size. β-catenin reduced apoptosis in cardiac fibroblasts and cardiomyocytes and enhanced survival and Bcl-2 expression. β-catenin also elevated the proportion of cells in the S phase, with upregulated cyclin D1 and E2 expression in cardiac fibroblasts and cardiomyocytes.^7^ Song et al. identified that theacrine could mitigate myocardial fibrosis following myocardial infarction through the sirtuin3/β-catenin/peroxisome proliferator-activator receptor γ pathway in mice lacking estrogen.^24^ These reports suggest that β-catenin is associated with the development of MI and exerts a cardioprotective function. Here, the authors observed that β-catenin was downregulated in the cardiac tissue of MI rats and in H_2_O_2_-treated cardiomyocytes, compared with non-MI rats and non-treated cells. Overexpression of β-catenin ameliorated infarct size and pathological changes and reduced the number of apoptotic cells in cardiac tissue from MI rats. At the cell level, the authors found that β-catenin overexpression increased cell viability and reduced apoptosis in H_2_O_2_-treated cardiomyocytes, indicating a cardioprotective role for β-catenin during MI development.

β-catenin acts as an upstream modulator of YAP in tumor cells.^8^ Ramjee et al. found that mice lacking epicardial YAP and TAZ, two key effectors of the Hippo pathway, display severe myocardial fibrosis and pericardial inflammation after MI, leading to cardiomyopathy and death.^11^ Mia et al. showed that YAP and TAZ expression is upregulated in macrophages undergoing reparative or pro-inflammatory phenotype alterations. Genetic deletion of YAP and TAZ resulted in inhibited pro-inflammatory and elevated reparative responses. In contrast, YAP activation was capable of elevating proinflammatory and inhibiting reparative responses. YAP and TAZ deletion in macrophage polarization resulted in decreased hypertrophy and fibrosis, as well as enhanced angiogenesis, thereby ameliorating cardiac function following MI.^10^ Another study by Lin et al. also found that YAP activation following MI preserved heart function and decreased infarct size.^25^ In the present study, it was observed that induction of MI caused deactivation of YAP, while β-catenin overexpression led to its reactivation in the cardiac tissue of MI rats, suggesting that β-catenin expression is positively associated with YAP activity; this was also confirmed by the *in vitro* data. In H_2_O_2_-treated cardiomyocytes, YAP reactivation by MA-5 administration resulted in reduced apoptosis, whereas in β-catenin-overexpressing and H_2_O_2_-treated cardiomyocytes, CIL56 treatment impaired cell viability and promoted cell apoptosis. These data suggest that YAP, which is positively regulated by β-catenin, can also preserve heart function during MI progression. However, a limitation of the present work is that the authors did not elucidate the function of YAP in MI rats.

In summary, β-catenin expression and YAP activity were significantly downregulated in MI rats. Overexpression of β-catenin facilitates the proliferation and suppresses the apoptosis of impaired myocardial cells by activating YAP signaling. LV-CTNNB1 transduction induces myocardial cell recovery after MI in an animal model. Therefore, elevating the expression of either β-catenin or YAP in MI patients to some degree may partially alleviate the progression of acute MI. The novelty of this study is the finding that the β-catenin/YAP axis may be a target for the treatment of acute MI.

## CRediT authorship contribution statement

**Haofei Kang:** Conceptualization, Data curation, Formal analysis, Investigation, Methodology, Project administration, Supervision, Visualization, Writing – original draft. **Weiwei Jiang:** Conceptualization, Data curation, Formal analysis, Investigation, Methodology, Supervision, Visualization, Writing – original draft, Writing – review & editing.

## Conflicts of interest

The authors declare no conflicts of interest.
